# The challenges of estimating the human global burden of disease of antimicrobial resistant bacteria

**DOI:** 10.1016/j.mib.2020.09.013

**Published:** 2020-10

**Authors:** Susanna J Dunachie, Nicholas PJ Day, Christiane Dolecek

**Affiliations:** 1 Centre for Tropical Medicine and Global Health, Nuffield Department of Medicine, University of Oxford, UK; 2 Mahidol-Oxford Tropical Medicine Research Unit, Faculty of Tropical Medicine, Mahidol University, Bangkok, Thailand

## Abstract

Estimating the contribution of antimicrobial resistance (AMR) to global mortality and healthcare costs enables evaluation of interventions, informs policy decisions on resource allocation, and drives research priorities. However assembling the high quality, patient-level data required for global estimates is challenging. Capacity for accurate microbiology culture and antimicrobial susceptibility testing is woefully neglected in low and middle-income countries, and further surveillance and research on community antimicrobial usage, bias in blood culture sampling, and the contribution of co-morbidities such as diabetes is essential. International collaboration between governments, policy makers, academics, microbiologists, front-line clinicians, veterinarians, the food and agriculture industry and the public is critical to understand and tackle AMR.

## Highlights

• Current estimates of the global burden of AMR are limited by lack of data.
• Choice of methodological approach for calculating AMR burden impacts on estimates.
• Patient-focussed surveillance of drug-resistant infection is a priority.
• International collaboration to build sustainable AMR surveillance is essential.

**Current Opinion in Microbiology** 2020, **57**:95–101

This review comes from a themed issue on **Antimicrobials**

Edited by **Iruka N Okeke** and **Audrey R Odom John**

For a complete overview see the Issue and the Editorial

Available online 2nd November 2020

**https://doi.org/10.1016/j.mib.2020.09.013**

1369-5274/© 2020 The Author(s). Published by Elsevier Ltd. This is an open access article under the CC BY license (http://creativecommons.org/licenses/by/4.0/).

## Introduction

Antimicrobial resistance (AMR) creates a significant health and economic burden for society [1], and a world where bacterial infections are untreatable due to AMR is a growing threat. Potential drivers of AMR include inappropriate use of antibiotics [2,3,4^•^], poverty [5, 6, 7], poor sanitation [8^•^], international travel [9,10], and increased healthcare interventions [4^•^] for an increasingly frail population. Further research is needed to determine the relative contribution of each potential driver to the burden of disease caused by AMR, and to develop effective strategies for intervention. We have yet to discover the impact of COVID-19 on global AMR rates. High antimicrobial use in suspected COVID-19 cases [11], disruption of AMR surveillance programmes [12], and economic decline as a result of the pandemic are risks for worsening AMR, but in some settings higher emphasis on infection control alongside decreased travel could decrease transmission of resistant bacteria [13]. Estimating the global burden of AMR is desirable for decisions on resource allocation, driving research priorities, evaluation of interventions, comparisons between countries, and comparisons with other diseases [14,15]. ‘What gets measured gets managed’ is a quote from the management guru Drucker [16], and in order to tackle the rapidly increasing threat of AMR, it is essential to understand the global burden of disease.

## Previous estimates of the burden of AMR, and current initiatives

In recent years a number of landmark estimates of the burden of AMR nationally and regionally have been published, including estimates from the US Centre for Disease Control and Prevention (CDC) [17], the European Centre for Disease Prevention and Control (ECDC) [18,19^••^] and from Thailand [20,21]. However, as each study uses different case definitions, data sources and methodology to calculate disease burden, direct comparison of these estimates is not possible. In 2014, the UK government commissioned the economist Jim O’Neill to undertake the ‘*Review on AMR*’; the much quoted report estimated that around 700 000 deaths each year globally may occur from infections with AMR bacterial infections, including multidrug-resistant and extensively drug-resistant tuberculosis [22]. This review has provided much wanted ballpark figures for policy makers, but it is widely acknowledged that the estimates, which were based on extrapolation of European and US data, require extensive revision [23,24,25^••^].

Many outstanding surveillance networks for AMR exist in low-income and middle-income countries (LMICs) (reviewed in Ref. [26]), but recent programmes seek to widen and deepen surveillance. The Global Antimicrobial Resistance Surveillance System (GLASS) launched by the World Health Organization (WHO) is developing a standardised approach to the collection, analysis and sharing of AMR data at a global level [27]. This initiative supports the establishment of national AMR surveillance systems and will greatly progress the collection of reliable and comparable data to allow monitoring of trends and evaluation of future interventions. In parallel, the Fleming Fund, a UK Aid programme by the UK Department of Health and Social Care is supporting LMICs to improve the surveillance of AMR and generate data that can be shared nationally and globally [28]. ResistanceMap [29] is a global database of national and subnational data on antimicrobial use and AMR which represents an extensive resource for prediction [30].

In addition, pioneering studies are yielding high-quality data from prospective studies in complex multi-national settings, including the BURDEN Study for MRSA in Europe [31], the PANORAMA Study for carbapenem resistance in LMIC settings [32^•^], and the BARNARDS Study in neonates in LMIC settings [33].

An international collaborative effort is now underway to map and estimate the global burden of AMR [24], with the Global Research on AntiMicrobial resistance (GRAM) Study combining Oxford University’s Tropical Medicine and Big Data Institute expertise with the Global Burden of Disease (GBD) framework at the Institute of Health Metrics and Evaluation at the University of Washington. This collaboration aims to produce the first estimates of the global burden of AMR by 2021, with incorporation into the GBD framework by 2022. The accuracy of these estimates will be limited by the challenges outlined here, but such work can form the foundation for future iterations as further data and knowledge of bias becomes available. It is widely acknowledged that this project faces a number of considerable challenges, and this review will outline these and potential solutions.

## Challenge 1: the unique characteristics of AMR

Bacterial AMR is a unique problem, for a number of reasons, and simply transferring approaches that have successfully estimated the burden of other infections such as malaria and HIV is likely to be insufficient. First of all, AMR is not a disease in itself, and isolation of bacteria from a person may represent commensal carriage rather than disease; it is drug-resistant infection (DRI) that creates the disease burden. A large number of bacterial species cause DRI, each species can develop AMR to several antibiotics, and AMR genes can spread between bacterial species via plasmid transfer (e.g. the spread of carbapenemases among *Enterobacteriaceae* [34]), rather than being species-specific. Estimates of disease burden need a decision on which bacteria/DRI to include. In the microbiology laboratory, the protocol for identifying and undertaking antimicrobial susceptibility testing (AST) is different for each bacteria, meaning that competency takes time and resource to develop. Infections with bacteria such as *Staphylococcus aureus* and *Escherichia coli* can lead to multiple clinical syndromes (e.g. urinary tract infections, pneumonia etc), and these bacteria are not included in GBD estimates to date [35]. The main cause of morbidity and death in bacterial disease is sepsis, which is notoriously difficult to define and has undergone changes in definition [36]. Until recently sepsis was excluded from the GBD framework [35], with new progress requiring an estimation of the contribution of sepsis to mortality from each disease in the GBD framework [37]. There is the need to consider potential threat, for example posed by *mcr-1* mediated colistin resistance [38], as well as the current disease burden caused by more prevalent AMR determinants. Finally, the drivers, distribution and solutions for AMR are likely to be very different in low-income settings compared to high-income countries, and it is likely that different approaches are needed for different regions of the world [6,39].

## Challenge 2: data related

**Figure 1:**
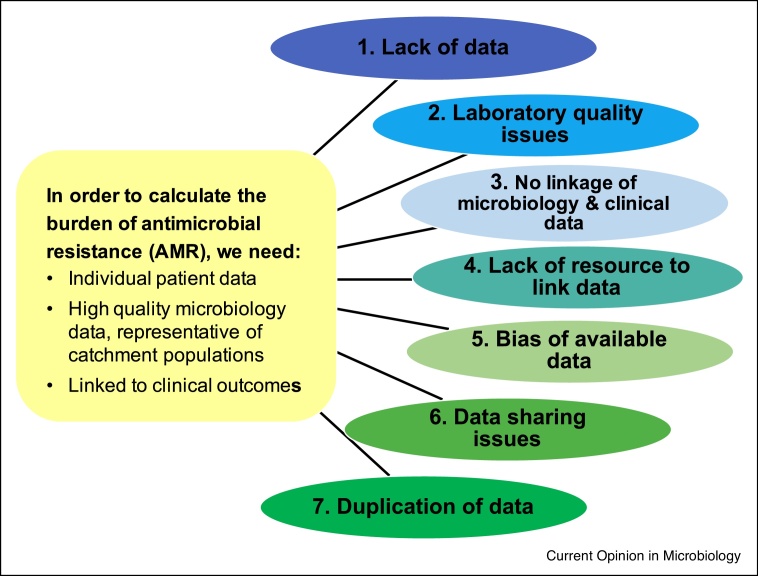
Overview of data-related challenges. Seven types of data-related challenges for estimating the global burden of antimicrobial resistance are shown. See text for further details.

There is general consensus that the biggest challenges for estimating the global burden of AMR are the lack of good quality microbiological data, the fact that existing microbiological data is not linked to clinical data including outcomes, and the knowledge that available data is often not representative of the general population. There are several reasons for this (Figure 1):

(1) **Lack of data.** For much of the global south including most of Sub-Saharan Africa, there are minimal facilities for microbiological culture, and even when access to microbiology laboratories is available, there is a lack of ‘culture to culture’ amongst front-line clinical staff.
(2) **Laboratory quality issues.** Where microbiology data exists, there may be concerns about the quality of the data, and information to evaluate quality such as the use of bacterial control strains, participation in external quality assurance schemes (EQAs) and international laboratory accreditation is often unavailable [40]. Standardisation of quality reporting is much needed [41^•^].
(3) **No linkage of microbiology and clinical data.** Good quality microbiology data may exist but without the linked patient metadata (such as clinical syndrome, co-morbidities and mortality outcome) that is required to estimate burden. A more patient-centred focus on data collection is needed.
(4) **Lack of resource to link data.** Although there may be the potential to link microbiology data to patient hospital records to obtain this vital metadata, the resources, working relationships, political support and permissions are not in place.
(5) **Bias of available data.** Good quality microbiology data is rarely representative of the underlying population, and such biases are very difficult to quantify. In resource-restricted settings where the costs of blood culture is borne out-of-pocket by the patient’s family, blood culture sampling is much more likely to be available in cities and in the private sector, and often restricted to complex hospitalised patients not responding to first-line antibiotics. Additionally, access the antibiotics is not uniformly distributed, and in many LMIC settings there is a steep drop-off between antibiotic overuse and lack of access to antibiotics [42].
(6) **Data sharing issues.** High quality datasets of bacterial isolates linked to patient metadata may exist in hospital systems, research institutes, public health and commercial systems, but these are usually not rapidly sharable: patient preferences, confidentiality, the ethics of data sharing, local and national sensitivities (such as fear of being exposed on a map as having high rates of AMR), and conflicts with ongoing research projects of the data custodians are all important factors.
(7) **Duplication of data.** Finally, given the scarcity of data, it is important to ensure there is no significant duplication of data used for analysis, as one dataset can be published several times or shared with multiple agencies.

## Challenge 3: choice of methodology

The next challenge for producing reliable estimates of the global burden of AMR lies with the choice of methodology, and this has been reviewed recently [25^••^]. In brief, given an ideal dataset of high quality, representative microbiological data linked to patient data, there remains a choice of how to measure the disease burden. Excess length of stay, disability-adjusted life years (DALYs), years of life lost and annual mortality are all potential metrics, with several approaches for calculation. The GBD framework uses single underlying cause of death from death certificates for mortality estimates [35] — this has the advantage of being easily understood, can utilise international death certificate data, and avoids double-counting of deaths. However this approach is not well suited for AMR estimation, where sepsis may be a key contributor to death but an underlying medical condition such as diabetes or ischaemic heart disease will be reported as the cause of death in national statistics. Another approach is ‘all cause’ mortality, which has been used for cohort studies [21] and by the UK Office of Statistics for measuring death from MRSA [43]. This approach is inclusive and comprehensive, but is also labour intensive, requires studying subpopulations then extrapolation, and measures association only without ascertaining causality.

The counterfactual approach, where the number of deaths occurring in the context of AMR are compared with a theoretical alternative scenario, deals with multi-factorial causes and is closer to addressing causality, but requires high quality, granular datasets and relies on modelling, such that the validity of the assumptions are crucial. A key issue when assessing the AMR burden is which comparator to use. For example, should we compare deaths in people with MRSA bacteraemia with deaths in people with sensitive *S. aureus* (MSSA) bacteraemia? This assumes that MRSA bacteraemia replaces MSSA in a simple 1:1 relationship, but there is some evidence that this may not be the case and instead MRSA and MSSA may show independent epidemiological dynamics [44,45]. Therefore the comparator for the counterfactual model could be no infection at all (with further issues around how to generate this comparator group). The counterfactual approach allows AMR to be treated as a risk factor for death in the context of multi-morbidity, and it may be that calculation of the relative risk of death for drug-resistant infection versus both drug-susceptible infection and no infection would provide credible limits for the estimate.

The best methodological approach for estimating the burden of AMR may differ for community-acquired versus hospital-acquired infection, and high-income countries versus LMICs. For better understanding of the global burden of AMR, there is a place for both detailed, ground-up approaches of estimating burden in one region at a time, where biases and limitations can be clearly understood and analysed, in parallel with birds-eye ‘top down’ approaches as used by the GRAM Study.

## Challenge 4: integration with one health

The problem of AMR in humans does not exist in isolation, and needs to be considered in the wider context of AMR in wild animals, farmed livestock, agriculture and the wider environment (reviewed in Ref. [46]). The environment can become contaminated with both AMR bacteria and antibiotics through human activities including human sewage disposal [47], hospital waste waters [48,49], farm animal effluent [50,51], aquaculture (farming of fish and seafood) [52], industrial waste [53] and use of antibiotics in agriculture [54] and livestock [55]. Further research and surveillance is needed to quantitate the problem and understand how to improve sanitation and wastewater disposal. The contribution of antibiotic use in animals to the human AMR burden is not fully understood. Recent studies have suggested limited sharing of bacterial strains and resistance genes between animals and humans in the UK [56^•^,^57^], but this may differ in LMIC settings, and is certainly important for food-borne bacteria such as non-typhoidal salmonella [58].

## Challenge 5: special circumstances

Obtaining high quality data and undertaking estimates of global burden is already challenging for bacteria that cause acute disease and are commonly cultured in microbiology laboratories, but additional issues are present for some other bacteria which have developed clinically important resistance to antimicrobials. Multidrug resistance is an enormous global threat for *Mycobacterium tuberculosis* (TB), and estimating disease burden has distinct challenges. TB is a chronic infection which takes several weeks to culture, and latent infection is a key feature. If bacteria have been isolated from multiple clinical samples during an acute infection caused by bacteria such as *E. coli* or *S. aureus*, it is reasonable to use the first isolate for analysis. However, in TB important resistance may develop during treatment, and therefore it is important to consider susceptibility when *MTb* is isolated several weeks into treatment (treatment failure). Global TB reporting is good compared to other bacterial infections, thanks to WHO’s global TB reporting system [59], but point prevalence surveys indicate that some countries still have low case detection rates (e.g. 24% for Nigeria in 2018 [60]), and these countries are the focus of current enhanced surveillance strategies.

Drug resistant gonorrhoea is an alarming concern, with a recent report of a patient with an *Neisseria gonorrhoea* isolate resistant to both ceftriaxone and high-level azithromycin [61], but gonorrhoea remains a Cinderella infection in terms of attention and resources allocated. The majority of the world’s management of sexually transmitted infections is syndromic, meaning patients are treated empirically without a specific diagnosis, or PCR-based detection is increasingly used meaning susceptibility testing is not performed. In addition, surveillance and research are typically conducted in high risk subpopulations only, meaning that the generalisation to larger populations is problematic. Support of key sentinel surveillance centres and championing of the WHO’s Gonococcal Antimicrobial Surveillance Programme (GASP) [62] and other initiatives is vital in order to keep ahead of the spectre of incurable gonorrhoea.

## Future directions

With the current lack of high quality, patient-level microbiology data linked to clinical outcomes that is truly representative of populations around the world, making accurate estimates of the global burden of AMR is extremely difficult. There is also debate about the best methodical approach to use, and how to overcome the inevitable biases in the available data. Nevertheless, we live in unprecedented times for AMR surveillance. There is genuine enthusiasm from a diverse range of government, academic, commercial, charitable, public and funding agencies to work together to support surveillance and find intelligent solutions for data re-use and sharing. Exemplar programmes for high quality national AMR surveillance are being developed, for example at the National Health Laboratory Service, South Africa [63] and at the Indian Council for Medical Research [64]. International collaboration is vital to develop accurate and sustainable surveillance. The Surveillance and Epidemiology of Drug Resistant Infections Consortium (SEDRIC) is a Wellcome-funded consortium working to transform strategy into action for AMR surveillance, and is supporting initiatives to develop real-world surveillance systems which include the essential components for burden estimation such as ACORN [65]. The next few years will see progress from GLASS and Fleming-funded networks which work at grass-roots level to build capacity frameworks and local expertise.

Ultimately, we believe that a patient-centred approach rather than the current focus on microbiology laboratories is required, and we need approaches that support the development of expert skills for both data collection and local data analysis to address the key questions in each community, build the knowledge base, and allow bench-marking of good practice and evaluation of new interventions (Box 1). Research initiatives to define the burden of AMR are driving a market for good quality data, and are helping to elucidate what we don’t know, which gives opportunity for scientists around the world to work together to fill the knowledge gaps and share solutions. The global crisis of COVID-19 has emphasised the need for international co-operation, and it is imperative that the next decade sees considerable growth in our understanding of the burden of AMR, and in defining the tools necessary for control.

Box 1 **Top 10 actions to improve estimates of global burden of AMR**

1 Championing of patient-centred surveillance of drug-resistant infection
2 Raising the status of microbiology as a medical speciality worldwide
3 Building capacity for in-country analysis and leadership in LMIC settings
4 Defining the minimum set of key variables required for estimating AMR and developing data capture systems
5 Creating sampling strategies to define and overcome biases of available blood culture data
6 Development of systems to define and improve the quality of laboratory microbiological data
7 Investment in research to understand the role of antibiotic use and AMR in animals and in the environment
8 Social sciences research to understand antimicrobial usage and health seeking behaviour
9 Supporting data re-use to allow comparison of methodology
10 Stakeholder engagement to understand the uses and limits of AMR burden estimates

Alt-text: Box 1

## Conflict of interest statement

Nothing declared.

## Funding

This work was supported by the Fleming Fund at the Department of Health and Social Care, UK, the 10.13039/100010269Wellcome Trust (209142/Z/17/Z), and the 10.13039/100000865Bill and Melinda Gates Foundation (OPP1176062).

## References and recommended reading

Papers of particular interest, published within the period of review, have been highlighted as:

• of special interest
•• of outstanding interest
